# Performance enhancement of single layer organic light-emitting diodes using chlorinated indium tin oxide as the anode

**DOI:** 10.1039/c7ra13355c

**Published:** 2018-03-21

**Authors:** Zhenxuan Wu, Zhenlin Yang, Kai Xue, Chunchun Fei, Fei Wang, MinNan Yan, Hongmei Zhang, Dongge Ma, Wei Huang

**Affiliations:** Institute of Advanced Materials (IAM), Nanjing University of Posts & Telecommunications (NUPT) Nanjing 210023 P. R. China iamhmzhang@njupt.edu.cn; Key Laboratory for Organic Electronics and Information Displays, Jiangsu Key Laboratory for Biosensors, Nanjing University of Posts & Telecommunications (NUPT) Nanjing 210023 P. R. China; Jiangsu National Synergetic Innovation Center for Advanced Materials (SICAM), Nanjing University of Posts & Telecommunications (NUPT) Nanjing 210023 P. R. China; Institute of Polymer Optoelectronic Materials and Devices, State Key Laboratory of Luminescent Materials and Devices, South China University of Technology Guangzhou 510640 P. R. China

## Abstract

Effective green single-layer organic light-emitting diodes (OLEDs) are reported with *fac*-tris(2-phenylpyridine)iridium [Ir(ppy)_3_] as a dopant and chlorinated indium tin oxide (Cl–ITO) as a transparent anode. The work function of the chlorinated ITO is manipulated to be ∼5.3 eV from ∼4.7 eV for bared ITO. The improvement in anode workfunction allows the direct hole injection into the HOMO of the phosphorescent dopant. As a result, the green phosphorescent OLEDs with simple single layers can deliver a current efficiency (CE) and external quantum efficiency (EQE) as high as 33.48 cd A^−1^ and 10.1%, respectively.

## Abstract 2

Effective green single-layer organic light-emitting diodes (OLEDs) are reported with *fac*-tris(2-phenylpyridine)iridium [Ir(ppy)_3_] as a dopant and chlorinated indium tin oxide (Cl–ITO) as a transparent anode.

## Graphical abstract

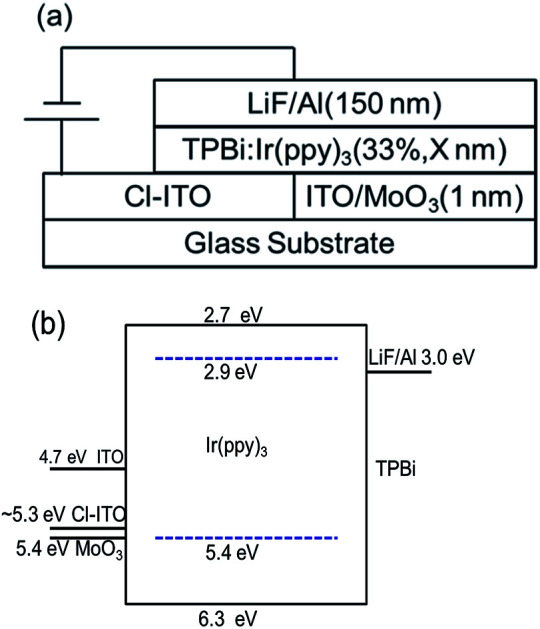

## Introduction

Phosphorescent OLEDs have become practical to use in the field of display and solid-state lighting because of their high internal quantum efficiency compared to conventional fluorescent OLEDs.^1,2^ In order to achieve high efficiency, it is essential to confine the triplet excitons within the emitting layer (EML), which requires that the host material has a higher triplet energy relative to emissive phosphors. Employing hole and electron blocking layers (HBL and EBL) to confine the holes and the electrons inside the EML are also necessary.^3–5^ In addition, the insertion of other functional layers including a hole injecting layer, a hole transport layer and an electron transport layer can further facilitate charge carrier injection and transport, and prevent triplet exciton quenching at the EML/HBL or EBL/EML interfaces. In addition, a multilayer structure may balance the charge carrier transport in the EML to enhance the device’s lifetime as well as its efficiency.^6–8^ However, such complicated device architectures inevitably increase the manufacturing complexity and the production cost. Therefore, a simplified device structure is important to reduce costs in practical applications for mass production. In the past two decades, simplified devices have been extensively studied.^16,34,45^ So far, the maximum reported current efficiency of green single-layer OLEDs can be up to 60 cd A^−1^.^16^ However, the devices showed a distinct efficiency roll-off.

A number of strategies have been developed to construct the simplified devices, including the utilization of bipolar host materials^9,10^ or mixed host materials^11,12^ to balance the carrier transport or broaden the recombination zone. The excellent performance of single layer OLEDs could be due to both the broader exciton formation zone and the balancing of the charge carrier injection.^13^ Otherwise, as a result of unbalanced charge conditions, the recombination zone is always located close to the metal electrodes, thereby causing exciton quenching by the electrodes and reducing the device efficiency.^14,15^ Thus, it is necessary to enhance the carrier injection, then adjust the carrier balance. It was known that the highest occupied molecular orbital (HOMO) of commonly used materials in phosphorescent OLEDs is typically 5.6 eV to 6.3 eV,^16–18^ which is much higher than the work function of ITO (∼4.7 eV) usually used as an anode.^19^ The modification of the electrode/organic interface plays a critical role in reducing the charge injection barrier. Many methods aiming to modify the ITO anode surface to achieve efficient carrier injection have been reported.^20–22^ When ITO is used as an anode, poly(3,4-ethylenedioxythiophene):poly(styrenesulfonate) (PEDOT:PSS) is commonly used as the hole injection material in OLEDs.^23,24^ Nevertheless, its acidic and hygroscopic nature can chemically degrade the organic layer and shorten the lifetime of the device.^25^ High work function transition metal oxide materials, likewise, have been introduced as efficient anode modification layers.^26,27^ Helander *et al.* reported a multilayer OLED using ITO electrodes treated with *o*-dichlorobenzene (*o*-DCB) as the anode.^28^ The method could enhance the ITO surface work function and the work function of the ITO surface could be adjusted with a different treatment time, with the highest up to 6.13 eV.^29,30^ A Cl–ITO electrode is often used to prepare multi-layered OLEDs.^30–33^ It is interesting to find that the work function of ITO can be tuned according to the number of Cl radicals from a halogenated solvent, the molecular structure and the duration of ultraviolet (UV) treatment. This will better match the energy levels of the host or guest of the light-emitting layer without introducing any additional injection layers.^28^ As a consequence, a Cl–ITO anode greatly simplifies the device structure. To date, Cl–ITO as an anode has been used in single layer polymer light emitting diodes.^43,44^ The single-layer devices with Cl–ITO gave the maximum brightness of 16 773 cd m^−2^ and maximum luminance efficiency of 2.40 cd A^−1^.^43^ But single layer OLEDs with Cl–ITO as the anode are little reported. In addition, the dopant of the emitting layer usually has the capability for hole transport in the phosphorescent OLEDs. It was also reported that the dopant Ir(ppy)_3_ can be used for hole injection and hole transport^34^ and TPBi was used for host and electronic transport. Thus, only the emitting layers of two component phosphorescent OLEDs were fabricated with the following structure: Cl–ITO/TPBi:Ir(ppy)_3_/LiF/Al, where the chlorinated solvents used were chloroform, chlorobenzene and *o*-DCB, respectively. The effects of the various chlorinated solvents and the various treatment times on the performance of single layer OLEDs were studied. The relation between chlorinated solvents and UV treatment time with the number of Cl atoms in the chlorinated solvents and the bond dissociation energies of C–Cl bonds was investigated. The high CE of 33.48 cd A^−1^ and EQE of 10.09% were achieved *via* exposing ITO to *o*-DCB under UV radiation for 5 min.

## Experimental section

A single layer OLED with MoO_3_ was fabricated as well for comparison. The devices with the following structures were investigated:

(1) ITO/MoO_3_ (1 nm or 0 nm)/TPBi:Ir(ppy)_3_ (*X*%, *Y* nm)/LiF (0.7 nm)/Al (150 nm)

(2) Cl–ITO/TPBi:Ir(ppy)_3_ (*X*%, *Y* nm)/LiF (0.7 nm)/Al (150 nm)

**Fig. 1:**
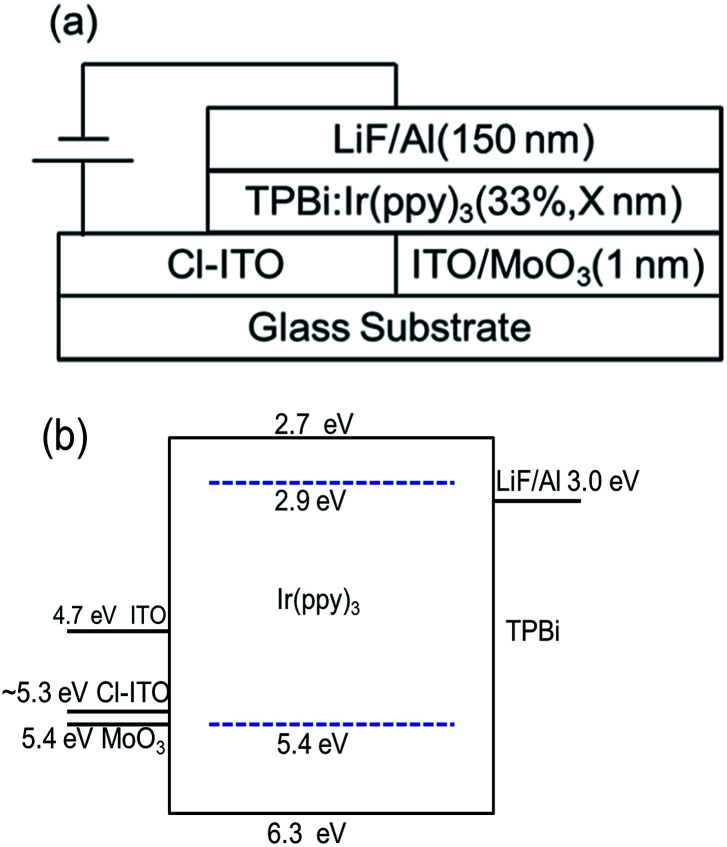
(a) The schematic device structure and (b) energy levels of the materials used in this study.

TPBi as a host and electron transport material, Ir(ppy)_3_ as a green dopant, and lithium fluoride (LiF) as an electron injection layer were purchased from commercial suppliers and used as received. The schematic device structure and energy levels of materials used are shown in Fig. 1.

### Device Fabrication

The commercial glass substrates precoated with an ITO layer with a sheet resistance of 10 Ω sq^−1^ were used. ITO glass substrates were successively rinsed with detergent, de-ionized water, acetone, and isopropyl alcohol. Each rinsing step was carried out in an ultrasonic bath (Shu mei KQ-300DE) for 15 min. The substrates were dried by a nitrogen flow. Cl–ITO was prepared by 5 minutes of UV ozone treatment of cleaned ITO, followed by UV treatment in a Pyrex Petri dish with 0.06 ml chlorinated solvents, varying the time of UV radiation during the chlorination process. Cl–ITO and ITO glass substrates were loaded into a vacuum evaporation chamber. MoO_3_, TPBi:Ir(ppy)_3_, LiF and Al were thermally evaporated onto glass substrates in turn under a pressure of ∼3 × 10^−4^ Pa. The deposition rates were 0.5 Å s^−1^, 1 Å s^−1^, 0.33 Å s^−1^, 0.05 Å s^−1^ and 5 Å s^−1^ for MoO_3_, TPBi, Ir(ppy)_3_, LiF and Al, respectively. The evaporation rates were monitored by a quartz oscillator system, and the film thickness was calibrated using a surface profiler (SQC-310, INFICON). The device area was defined as 16 mm^2^ by the overlap between the ITO and Al electrodes. The current–voltage-brightness characteristics were measured on a testing system consisting of a Keithley source measurement unit (Keithley 2400 and Keithley 2000) and a calibrated silicon photodiode. All devices were measured without encapsulation at room temperature.

## Results and discussion

**Fig. 2:**
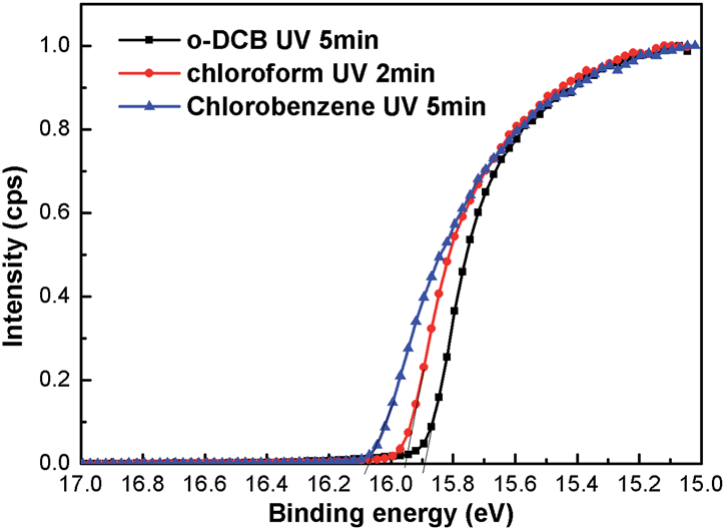
Secondary cut-off region of the UPS spectra of ITO anode substrates treated with *o*-DCB, chloroform and chlorobenzene, respectively.

Fig. 1 shows the device structure and energy levels of the materials used in this work. The work functions of ITO and MoO_3_ are 4.7 eV, and 5.4 eV,^35^ respectively. The work functions of Cl–ITO were measured by UPS (seen in Fig. 2) as 5.34 eV, 5.27 eV and 5.17 eV for *o*-DCB, chloroform and chlorobenzene, respectively. The HOMOs of TPBi and Ir(ppy)_3_ are 6.3 eV,^36^ and 5.4 eV,^37^ respectively. Based on the energy level alignment, the hole injection barrier from ITO/MoO_3_ to TPBi (0.9 eV) is high, while hole injection into Ir(ppy)_3_ is energetically favourable with a barrier of 0 eV. Therefore, holes are preferentially injected directly into the phosphorescent dopant rather than the host.^38^ The mechanism of influence of the chlorinated solvents on the performance of single layer OLEDs was investigated.

In single layer OLEDs, in the absence of a heterogeneous interface, the recombination of holes and electrons will be completed during transport, otherwise, the transfer of holes and electrons might form a dark current and decrease the efficiency of the devices.^34^ Furthermore, the recombination zone should be away from electrodes to suppress the exciton quenching effect when designing the single layer devices.^14,15^ Thus, the thickness and the dopant ratio of the emission layer should be optimized to control the recombination zone of holes and electrons.^13^

**Fig. 3:**
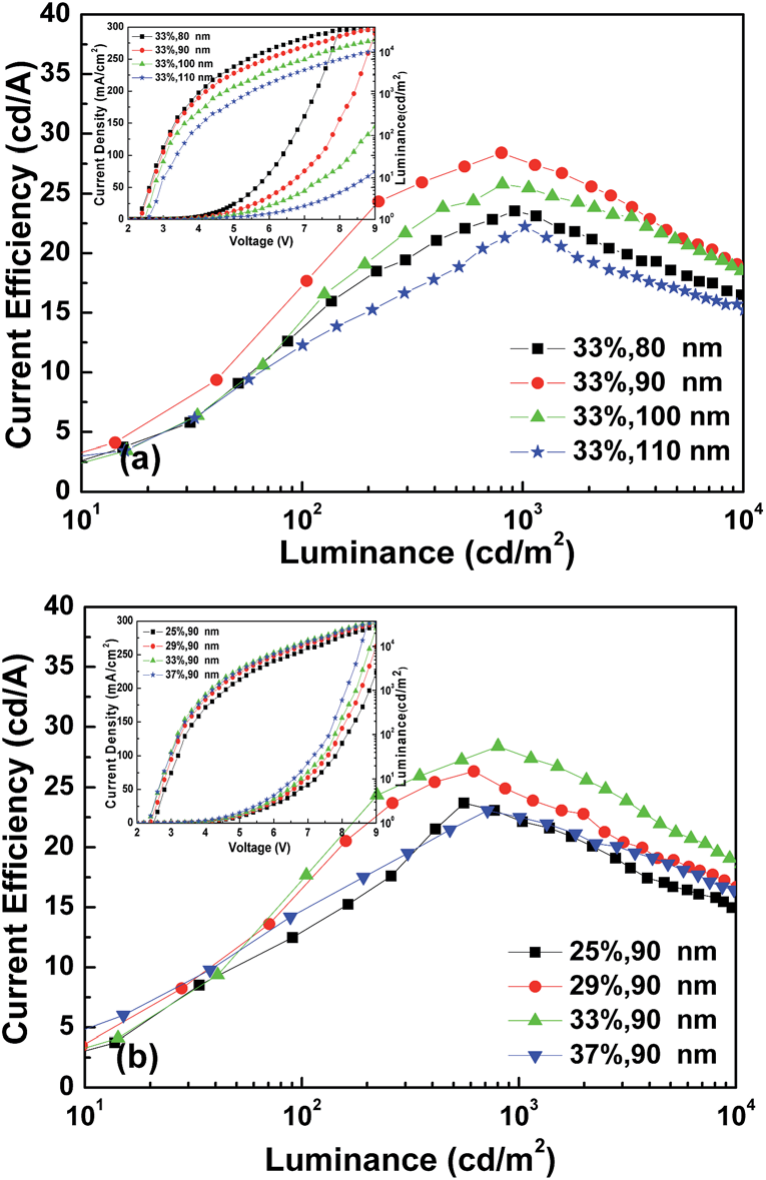
(a) CE–*L* characteristics of the devices ITO/MoO_3_ (1 nm)/TPBi:Ir(ppy)_3_ (33%, *Y* nm, *Y* = 80, 90, 100, 110 nm)/LiF (0.7 nm)/Al (150 nm). The inset gives the *J*–*L*–*V* of the devices. (b) JCE–*L* characteristics of the devices ITO/MoO_3_ (1 nm)/TPBi:Ir(ppy)_3_ (*X*%, 90 nm, *X* = 25, 29, 33, 37%)/LiF (0.7 nm)/Al (150 nm). The inset gives the *J*–*L*–*V* of the devices.

Taking into account the hole transport character of Ir(ppy)_3_, we chose an electron transporting material (TPBi) as the emission host. The devices were fabricated with a configuration of ITO/MoO_3_/TPBi:Ir(ppy)_3_ (*X*%, *Y* nm)/LiF (0.7 nm)/Al (150 nm) to optimize the thickness and dopant ratio of the emission layer. Fig. 3 shows the current density–luminance–voltage (*J*–*L*–*V*) and current efficiency–luminance (CE–*L*) characteristics of the OLEDs with MoO_3_ as the HIL.

The current efficiency of the device increases first and then decreases with the increase of the thickness and the dopant ratio. Based on the CE performance of the above devices, the optimum thickness and dopant ratio of the EML in the single layer OLEDs with MoO_3_ as the HIL were confirmed to be TPBi:Ir(ppy)_3_ (33%, 90 nm). Single layer OLEDs with Cl–ITO as the anode were then fabricated with the same thickness and dopant ratio of the EML. The devices investigated had the following structures:

Device A: ITO/MoO_3_ (1 nm or 0 nm)/TPBi:Ir(ppy)_3_ (33%, 90 nm)/LiF (0.7 nm)/Al (150 nm)

Device B: Cl-ITO/TPBi:Ir(ppy)_3_ (33%, 90 nm)/LiF (0.7 nm)/Al (150 nm)

The number of halogen atoms and the chemical structure of the molecule among halogenated solvents are different. The UV ozone treatment time *vs.* device performance was observed.

**Fig. 4:**
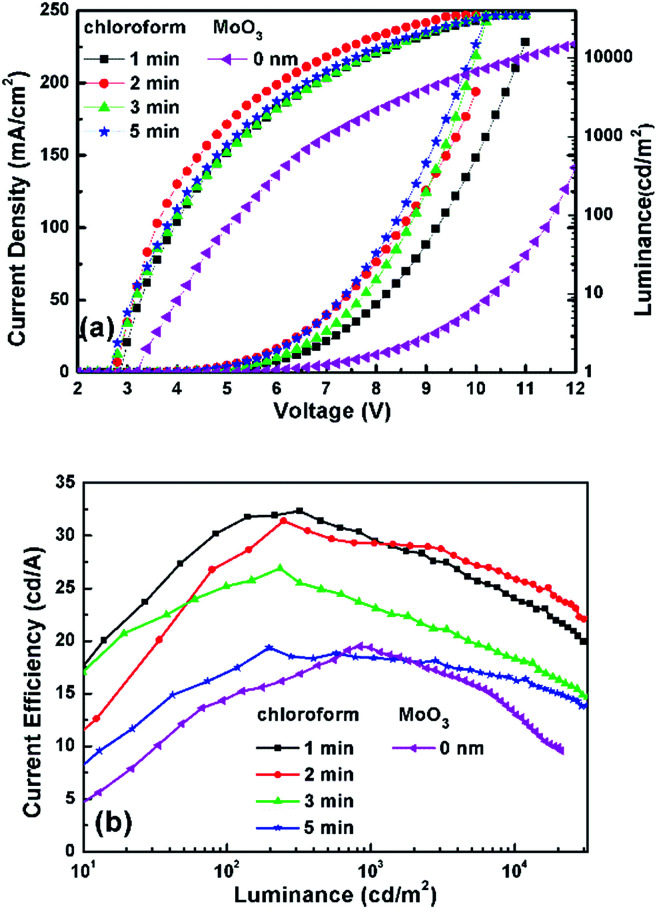
(a) *J*–*V*–*L* and (b) CE–*L* characteristics of devices with ITO treated by chloroform and only with UV.

In order to investigate the effect of solvent treatment time with UV ozone on the device performance, the treatment time (1, 2, 3, and 5 min) is optimized. Fig. 4(a), (b) and Table 1 show their current density–luminance–voltage (*J*–*L*–*V*) and current efficiency–luminance (CE–*L*) characteristics. The performance of the control device with a bare ITO anode is also shown in Fig. 4. All devices with chloroform solvent treatment show a lower turn on voltage (defined as the voltage required for a luminance of 1 cd m^−2^), with 3.2 V for the control device and 2.8–2.7 V for devices with Cl–ITO as the anode. This is because Cl–ITO exhibits a higher work function of 5.27 eV, as seen in Fig. 2. The hole injection barrier between the anode and the organic layer is reduced, which is more favourable for carrier injection. The current density increased with the increase of UV ozone treatment time from 1 min to 5 min. This could be attributed to the number of stable Cl–In dipoles which formed on the surface of ITO as treatment time with UV ozone increased. Fig. 4(b) shows that the efficiency of all devices increases in the initial 100 cd m^−2^. In single layer OLEDs, it is very sensitive to the balance of electrons and holes for carrier recombination because of the simplicity of the structure. The unbalanced charge injection and transport lead to a lower recombination probability and a shift of the carrier recombination zone to the electrodes, which results in a precarious efficiency.^39^ In addition, the efficiency of the devices first increases and then decreases gradually with the increase in UV ozone treatment time. It was found that the device with chloroform solvent treated for 2 min with UV ozone showed the maximum current efficiency of 31.39 cd A^−1^ under high luminance. The current density increases with treatment, implying that hole injection increases with the increase of the treatment time. However, the efficiency of the devices does not increase as the current density increases, which could be attributed to the increased hole injection which results in an unbalance of the charge carriers. The unbalanced carriers might form a dark current. This means that good performance of single layer OLEDs is related to both the improved hole injection and the balance of electrons and holes.

**Table 1:** The characteristics of devices with ITO treated by chloroform

UV time	Turn on (V)	CE (cd A^−1^)			
		Max	@100 cd m^−2^	@1000 cd m^−2^	@5000 cd m^−2^
1 min	2.8	32.35	31.75	29.49	26.14
2 min	2.7	31.39	28.68	29.28	27.54
3 min	2.7	26.89	25.20	23.12	20.00
5 min	2.7	19.35	17.48	18.38	17.27

Similarly, the influence of treatment time with UV ozone on the performance of the devices was investigated. Two groups of devices were treated by UV ozone with *o*-DCB or chlorobenzene solvent for 1, 3, 5, 7 min, respectively.

**Fig. 5:**
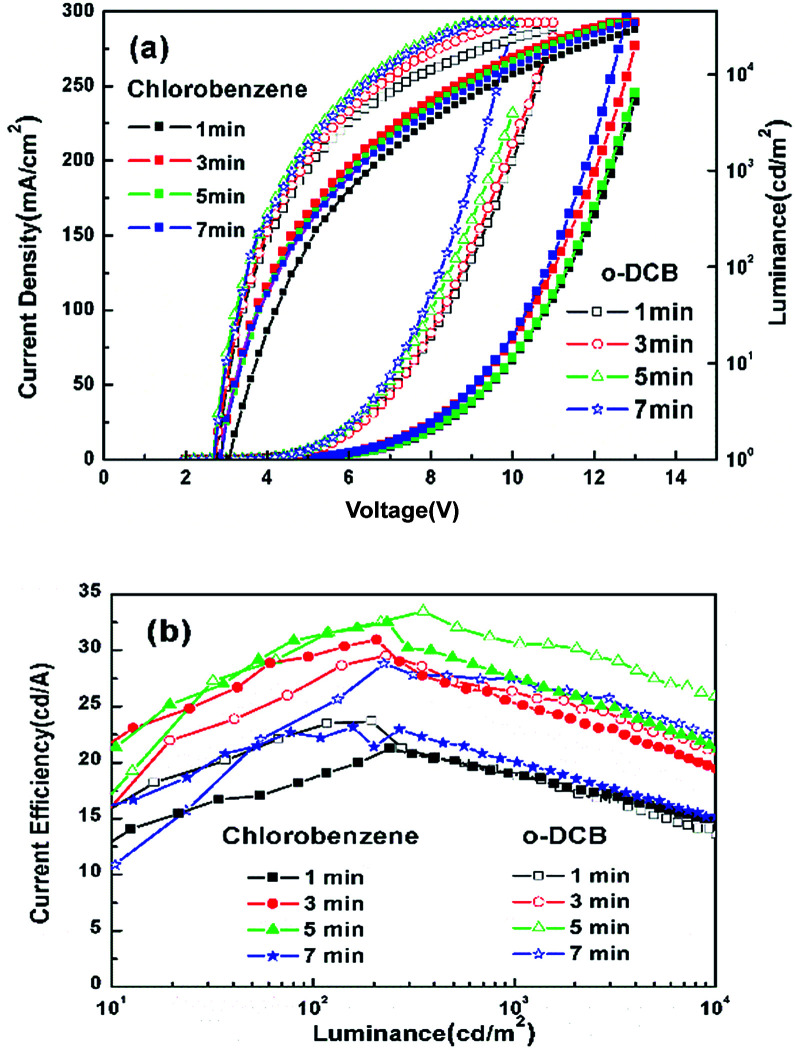
(a) *J*–*V*–*L* and (b) CE–*L* characteristics of devices with ITO treated by *o*-DCB and chlorobenzene.

Fig. 5 shows that the current density of devices treated with *o*-DCB is obviously higher than that of devices treated with chlorobenzene *via* exposing ITO to solvents under UV radiation for the same time. The turn-on voltage of the former is reduced from 2.9 V to 2.7 V, while that of the latter is reduced from 3.1 V to 2.8 V as the UV ozone treatment time increases. This could be associated with the number of Cl–In bonds. The efficiency of the two groups of the latter is reduced from 3.1 V to 2.8 V as the UV ozone treatment time increases. This could be also associated with the density of the Cl–In bonds. The efficiency of the two groups of devices first increased and then decreased with increased UV treatment time, and the best UV treatment time was 5 min, which was significantly higher than that for devices with chloroform treated for 2 min. The maximum CE of devices treated with *o*-DCB and chlorobenzene was 33.48 cd A^−1^ and 32.50 cd A^−1^, respectively.

**Fig. 6:**
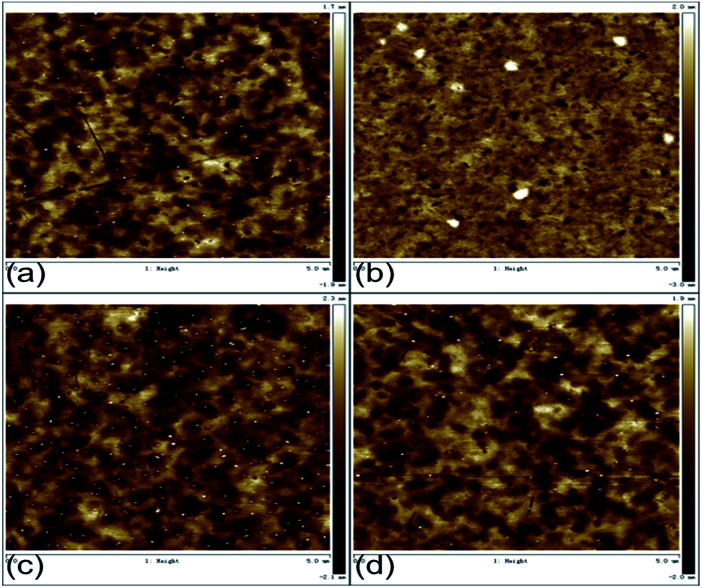
AFM of (a) ITO (UV 5 min), (b) Cl–ITO (chloroform, UV 2 min), (c) Cl–ITO (*o*-DCB, UV 5 min) and (d) Cl–ITO (chlorobenzene, UV 5 min).

In order to prove whether the improvement of the device performance is related to the surface morphology of the electrode, atomic force microscopy (AFM) image evaluation was carried out to observe the effects of chlorinated solvents and UV treatment time on the surface morphology of ITO. The scan area was 5 × 5 μm^2^. As shown in Fig. 6, we can see that the root-mean-square roughness (*R*_rms_) of ITO (UV 5 min), Cl–ITO (chloroform, UV 2 min), Cl–ITO (*o*-DCB, UV 5 min) and Cl–ITO (chlorobenzene, UV 5 min) were 0.580, 0.649, 0.668 and 0.623 nm respectively. It can be seen that there is no notable change compared to the pretreatment ITO. This proves that the improvement of the device performance should not be attributed to the change of the surface morphology.

**Fig. 7:**
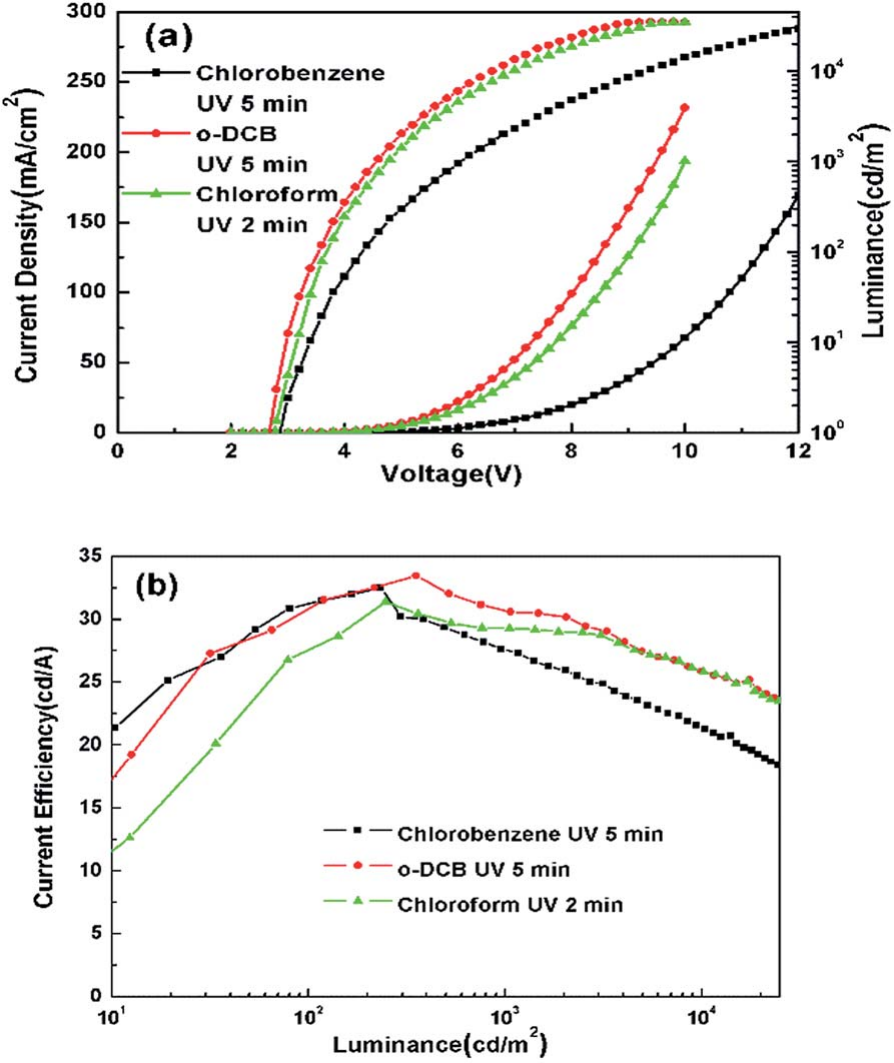
(a) *J*–*V*–*L* and (b) CE–*L* characteristics of the optimum devices with ITO modified by three kinds of chlorinated solvents.

The optimized performance for devices with various solvent treatments is shown in Fig. 7 and also summarized in Table 2. The optimum treatment time with *o*-DCB and chlorobenzene are both 5 min, but the *J*–*V*–*L* curves of the two devices are significantly different, while the optimum treatment time with chloroform is 2 min, and the *J*–*V*–*L* curves of the two devices treated with *o*-DCB and chloroform are similar. It was found that the performances of these devices depend on the number of Cl atoms in the chlorinated solvents, the molecular structure and the UV treatment time.

**Table 2:** The characteristics of devices with ITO modified by three kinds of chlorinated solvents

Chlorination solvent	UV time	*V*_on_ (V)	Current efficiency (cd A^−1^)			
			Max	@100 cd m^−2^	@1000 cd m^−2^	@5000 cd m^−2^
Chlorobenzene	5 min	2.9	32.50	31.48	27.66	23.56
*o*-DCB	5 min	2.7	33.48	31.55	30.59	27.46
Chloroform	2 min	2.7	31.39	28.68	29.28	27.54

The work functions of the ITO surface treated with three kinds of chlorinated solvents were studied by UPS (Fig. 2). The kinetic energy of the cut-off edge shifts to a higher energy after the Cl treatment. The enhancement of ITO work function is consistent with the number of stable Cl–In dipoles. The number of Cl–In dipoles is related to the number of Cl radicals liberated from the solvent displacing oxygen on the surface of the electrode.^28^ The number of Cl radicals liberated from the solvent can be explained by the bond dissociation energies. In chloroform, *o*-DCB and chlorobenzene molecules, the bond dissociation energies of C–Cl bonds are 318.8 kJ mol^−1^,^40^ 385.8 kJ mol^−1^^41^ and 399.6 kJ mol^−1^,^42^ respectively. Therefore, the Cl radicals in chloroform were most easily extracted from the solvent under the same UV ozone conditions, while Cl radicals were the most difficult to liberate from chlorobenzene. This is consistent with chloroform requiring UV ozone treatment for 2 min, while *o*-DCB UV requires ozone treatment for 5 min. Similarly, it reveals the reason why the current density of devices using *o*-DCB treatment is higher than that of devices using chlorobenzene treatment.

**Fig. 8:**
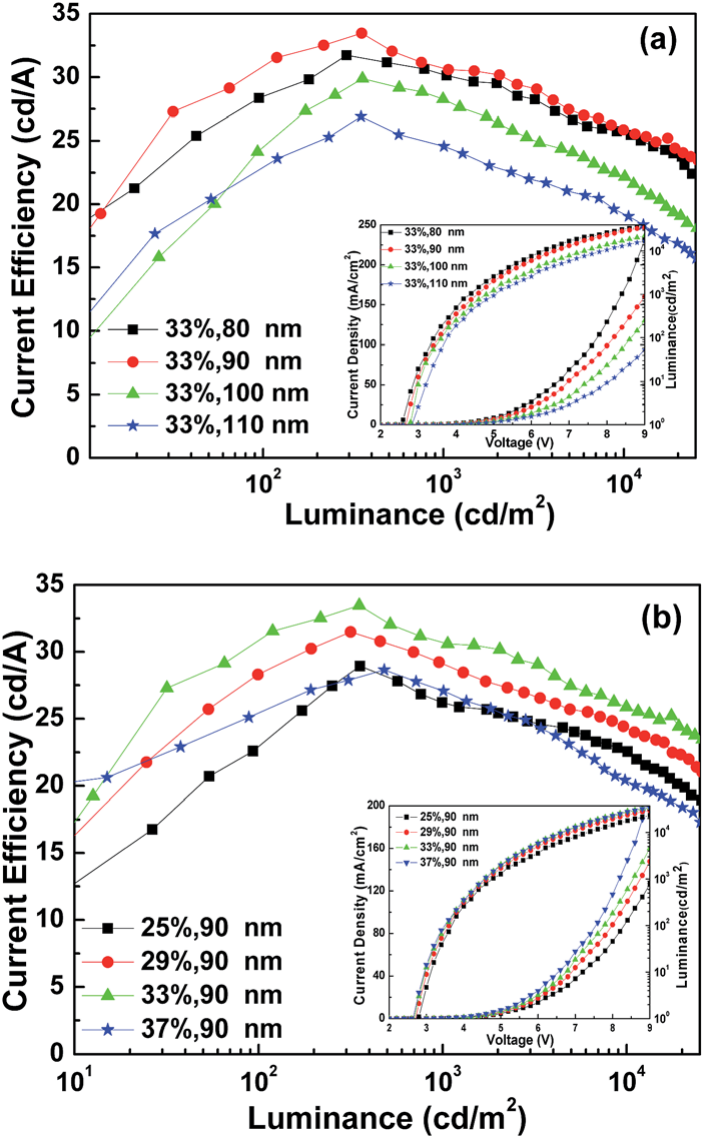
(a) CE–*L* characteristics of devices Cl–ITO (*o*-DCB, UV 5 min)/TPBi:Ir(ppy)_3_ (33%, *Y* nm, *Y* = 80, 90, 100, 110 nm)/LiF (0.7 nm)/Al (150 nm). The inset gives the *J*–*L*–*V* of the devices. (b) CE–*L* characteristics of devices Cl–ITO (*o*-DCB, UV 5 min)/TPBi:Ir(ppy)_3_ (*X*%, 90 nm, *X* = 25, 29, 33, 37%)/LiF (0.7 nm)/Al (150 nm). The inset gives the *J*–*L*–*V* of the devices.

The devices were fabricated with a configuration of Cl–ITO (*o*-DCB, UV 5 min)/TPBi:Ir(ppy)_3_ (*X*%, *Y* nm)/LiF (0.7 nm)/Al (150 nm) to optimize the thickness and the dopant ratio of the emission layer. The *J*–*V*–*L* and CE–*L* curves of the Cl–ITO based devices are shown in Fig. 8. The result shows a similar trend to that of the devices based on ITO/MoO_3_ shown in Fig. 3. When the thickness and the dopant ratio of the EML in the single layer are 90 nm and 33% respectively, the device performance is the best. The maximum CE of devices treated with *o*-DCB was 33.48 cd A^−1^. It should be noted that the devices exhibited a lower efficiency roll-off compared to the single-layer OLEDs and a higher CE compared to the polymer light emitting diodes in the literature.

**Fig. 9:**
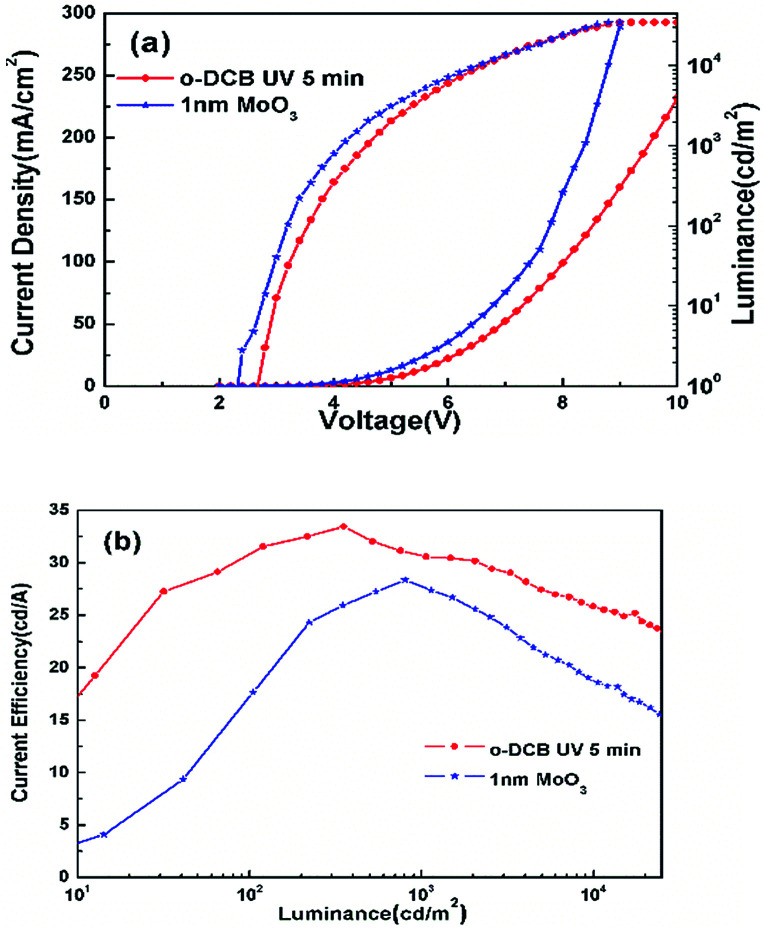
(a) *J*–*V*–*L* and (b) CE–*L* characteristics of the device with ITO treated by *o*-DCB and the device with 1 nm MoO_3_ as the ITO modified layer.

For comparison, the device ITO/MoO_3_ (1 nm)/TPBi:Ir(ppy)_3_ (33%, 90 nm)/LiF (0.7 nm)/Al (150 nm) was fabricated. Fig. 9 shows the comparison of the performance of the device using MoO_3_ as the HIL and the device treated using *o*-DCB which was the best chosen from the devices treated with three kinds of chlorinated solvents. It can be seen from Fig. 9 that the device using MoO_3_ as the HIL shows a higher current density than that of the device treated with *o*-DCB, while devices with Cl–ITO as the anode show a higher efficiency compared with the device with MoO_3_ as the HIL. This could be related to the charge balance. In order to explain the working mechanism of the charge balance, hole- and electron-only devices were designed with the following structures:

Device 1: Al/LiF (0.7 nm)/TPBi:Ir(ppy)_3_ (33%, 90 nm)/LiF (0.7 nm)/Al

Device 2: ITO/MoO_3_ (1 nm)/TPBi:Ir(ppy)_3_ (33%, 90 nm)/MoO_3_ (10 nm)/Al

Device 3: ITO (UV ozone treatment with *o*-DCB for 5 min)/TPBi:Ir(ppy)_3_ (33%, 90 nm)/MoO_3_ (10 nm)/Al

**Fig. 10:**
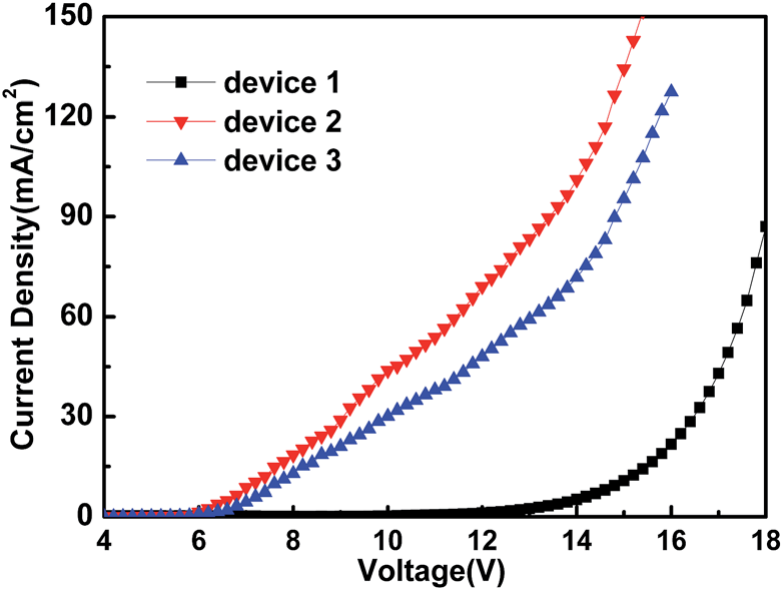
*J*–*V* characteristic of hole- or electron-only devices: device 1, device 2 and device 3.

The characteristic curves of *J*–*V* of the above devices are shown in Fig. 10. The *J*–*V* curve of device 3 (hole-only) compared with the *J*–*V* curve of device 2 (hole-only) appears to be nearer to the *J*–*V* curve of device 1 (electron-only). The result shows that the carrier injection of the device using MoO_3_ as the HIL was more unbalanced in comparison with the device with Cl–ITO as anode. The higher current could be attributed to a dark current rather than balancing the carriers. The charge balance and recombination is very important to achieve high efficiency.

## Conclusions

In summary, effective phosphorescent OLEDs in a single emission layer were fabricated based on ITO anodes by UV ozone treatment with chlorinated solvents. It is demonstrated that the improvement in the work function of the Cl–ITO anode depends on the number chlorine atoms. Since the bond dissociation energies of C–Cl bonds are material-dependent, the improvement in anode work function is sensitive to both solvent chemical structure and UV treatment time. The working mechanism of the charge balance is also explained through hole-only and electron-only devices. The result shows that control of the hole injection from the anode to balance the carriers and improve the recombination is necessary to attain high current efficiency in single-layer OLEDs.

## Conflicts of interest

There are no conflicts to declare.

## Supplementary Material
